# 

**Published:** 1808-06

**Authors:** 


### NEW MEDICAL PUBLICATIONS.

A Tract on the Nutriferous System in Men, Quadrupeds, and Birds, and in all Creatures which have Livers. By James Rymer, Surgeon, R. N. 2s. 6d.

Anniversary Oration, delivered March 8, 1808, before the Medical Society, on the General Structure and Physiology of Plants, compared with those of Animals, and the mutual Convertibility of their Organic Elements. By John Mason Good, Secretary to the Society. 2s.

The Principles of Botany and Vegetable Physiology. Translated from the German of D. C. Ludwic Willdenow, Professor of Botany at Berlin. 8vo. with ten plates, 10s. 6d.

An Introduction to Physiological and Systematical Botany. By James Edward Smith, M. D. F. R. S. &c. &c. President of the Linnæan Society. 8vo. with fifteen plates, 14s.

---

### To CORRESPONDENTS.

Mr. Walker's Tables of the Natural System of Medical Science, and of the First Principles of the Medical Art, will appear in our next Number.

---

END OF VOL. XIX.

### INDEX.

ABSCESSES, cafes of 184
Acetate of lead, efficacy of 69
Acid, on the oxalic 287
Acids of fweat, urine, and milk, on the 124
Adams, Dr. on vaccine inoculation 377
Adhefion, on the modern practice of 563
Æthiopia, peftilential winds in 116
Affufion, benefits of cold 175, 321
Africa, unhealthy fettlements in 114
Agrimony, defcription and ufes of 262
Air, changes induced in atmofpheric 371
—— on vibrations of the 403
Agues, remedy for 459
Akerly, Dr. on the cure of phthifis 224
Alder, Dr. on quarantines, &c. 113, 198
———— on proximate caufes of difeafes 334
Alkalies, decompofition of fixed 93, 190, 286
Alkohol, opium counteracted by 497
Alley's, Dr. cafe of diabetes mellitus 181
America, on the practice of medicine in 414
Anafarca, remedy for 73
Andrew's, Dr. on the ftate of the ifland of Madeira 19
Antivaccinift candour, on the 426
Anus, on a cafe of imperforate 420
Argentum nitratum, the ufe of 97
Arfenic, its ufe in eruption 206
——— on Fowler's folution of 369
——— in chronic rheumatifm 476
Articulations, cafe of the inflexibility of the 4
————, on the formation of foreign bodies in 84
Afthenia, obfervations on 324
Afthma, on fpafmodic 325
Atmofphere, influence of the 475
Auld's, Dr. cafe of bilious fever 106
Bandages, obfervations on 81
Banks, letter to Sir Jofeph 462
Bark, in meafles, on the ufe of 145
Barlow's, Dr. cafe of dyfphagia 248
Bartley, Mr. on an imperforate anus 420
Bath, on the ufe of the cold 111
Bayley, Mr. on inflexibility of the articulations 4
Beddoes, Dr. on fever 168
———— on medical abufes 462
Bellott's, Mr. cafe of fuppreffion of urine 227
———— obfervations on 523
Bilious fever, cafe of 106
Birch, Mr. extraordinary conduct of 557
Bladder, on a cafe of difeafed 13
Blood, oxids of iron in 191
Bones, on difeafes of the 79
BOOKS, MEDICAL, CRITICAL ANALYSIS OF—The Lectures of Boyer upon the Difeafes of the Bones, by A. Richerand, tranflated by Dr. Farrell, 79. A Treatife on Hernia, by Wm. Lawrence, 85. On Fever as connected with Inflammation, 168. Colloquia Anatomica, Phyfiologia, &c. auctore A. Robertfon, M. D. 178. A Letter on the Practice of Midwifery, by J. Boyd, *ib.* Edinburgh Medical and Surgical Journal, No. XIII, 179, 270, XIV 545. Treatife on Pulmonary Confumption, by J. Saunders, M. D. 263. Inquiry into the Changes of Atmofpheric Air, by Daniel Ellis, 371. Popular View of Vaccine Inoculation, by J. Adams, M. D. 377. Letter to Sir J. Banks on Abufes in Medicine, by T. Beddoes, M. D. 462. Hin s to Parliament, by W. Blair, 556. Attempt at a Syftematic Reform of the Modern Practice of Adhefion, by Samuel Young, 563.
BOOKS, NEW, MEDICAL ANNOUNCED Papers of the Medical and Chirurgical Society of London, 191. Modern Medicine, by Dr. Uwins, 192. Dr. Carpenter on the Structure and Function of the Eye, *ib.* The Medical Mentor, *ib.* Mr. Hill on Diftortions of the Spine and Limbs, 288. Mr. Good's Oration 331 Mr. Roberton on the Difeafes of Edinburgh, 384. Mr. Wadley's Letter on Vaccination, 384. Mr. Parkinfon's fecond volume of Organic Remains, 480. Mr. Paris's Compendium of Modern Chemiftry, *ib.* Mr. Carmichael's Effay upon Cancer, 576. Mr. Nicholfon's Chemical Dictionary, *ib.*
Botanical defcription of Britifh plants 71, 162, 257, 353, 455, 538
Bowels, on inflammation of the 183
Boys, Dr. on the practice of midwifery 178
Broom, defcription and ufes of 73
Broughton, Mr. on hydrophobia 491
Brown's, Dr. cafe of tetanus 423
Burn, remarkable cafe of 29

Burns, on the treatment of 17, 229
Burns, Mr. on lithotomy 271
Bufforah, unhealthy fituation of 114
Cader idris, a volcano 382
Cæfarean operation, cafe of 555
Calomel, its ufe in intermittent fevers 15
——— new method of preparing 190
——— mode of producing 382
Camphor, its ufe in fmall-pox 144
Cancer, efficacy of oil in 142
——— of the penis, fuccefsfully extirpated 270
Cantharides, efficacy of in fores 281
——— tetanus cured by 423
——— efficacy of in fluor albus 528
Caries, obfervations on 80
Carbon, obfervations on 373
Carmichael, Mr. on the cataract 310
Carwardine, Mr. on the action of the mufcles 289
Cafualties in London 96
Cataract, cafe of 101
——— obfervations on 310
Catarrh, on the treatment of 183, 187, 275, 323
——— remedy for 73
Catheter, fingular fhape of a 123
Cautery, on the ufes of the actual 429
Ceruffa acetata, effects of 8
Chamomile, virtues of 458
Children, an eruptive difeafe in 344
Chin-cough, obfervations on 184, 321
——— prevalence of 366
Chorea, on 326
Chronic intermittent, cafe of 53
Cinchona, its ufe in diabetes mellitus 181
———, effects of in gout 427
Cold affufion in fever, on 321
——— bath, utility of the 111
Colica pictonum, on the 12
Confultations, on medical 204
Confumption, cafes of pulmonary 47
——— efficacy of falivation in 20, 224, 506
——— on the treatment of pulmonary 263
——— ufe of emetics in pulmonary 337
——— on ftimulants in 510
——— cured by digitalis 511, 515
Contagion, obfervations on 115, 198
———, on difeafes fpread by 317
Controverfial communications, on 331
Cough, remedy for a catarrhous 73
——— on the chronic 276
Cow-pock inftitution, report of the Dublin 452
Crito, in reply to a practitioner 234
——— anfwer to 503
Croup, obfervations on 184, 321
Croup, cafes of 214, 321
Cullen, Dr. on meafles 147
Cynanche tonfillaris, on the 275, 282
Dandelion, defcription and virtues of the 166
Davies, Mr. on the ceruffa acetata 8
——— reply to 53
Davy, Mr. account of his experiments 93, 190
Dawfon, Mr. on a cafe of difeafed bladder 13
Derbyfhire practitioner, reply to a 234
——— his anfwer 503
Dew, remarkable noxioufnefs of 494
Diabetes mellitus, cafe of 181
Diarrhœa in meafles, cure of 146
Digitalis purpurea, efficacy of 420
——— its ufe in pulmonary confumption 511, 515
Difeafe, on the nofological characteriftics of 1
——— cafes of fecondary after meafles 133
Difeafes in London, monthly reports of 90, 92, 186, 189, 283, 285, 368, 370, 475, 477, 573
——— of Edinburgh, report of 182, 273, 278, 361, 470, 569
——— and cafualties in London in 1807 96
——— caufes of epidemic 115
——— on connected or correfponding 148
——— on the proximate caufes of 203, 334
Diffection of a morbid body 160
Diffections, obfervations on 154
Donovan's Mr. obfervations on cader idris 382
Dormice, obfervations on 383
Dropfy, obfervations on the 478
——— remedy for the 73
Drowning perfons, on faving 283
Dublin cow-pock inftitution, report of 452
Duck's Mr. cafe of crural hernia 501
Dyer's weed, defcription of 75
Dyfentery, diffections in 140
Dyfphagia, cafe of 248
Ear, anatomy and phyfiology of the 385
Edinburgh, reports of diseafes at 182, 273, 278, 361, 470, 569
——— clinical practice, on the 176
——— medical journal, account of the 179, 270, 545
——— defcription of 362
——— fchool of phyfic, on the 465
Egypt, on the difeafes in 114
Elecampane, virtues of 455
Electricity, chemical agencies of 93, 190
Ellis, Mr. on changes in the atmofpheric air 371

Ellis, Mr. remarks on the theory of 547

Emetics, their ufe in pulmonary confumption 337

Endemics, on 114

Epidemic difeafes, caufes of 115

——— on feafons attending 317

Epilepfy, acetate of lead adminiftered in 70

——— cafe of 305

Eruptions, ufe of arfenic in 206

——— remarkable cafe of 524

——— in children, cafe of 344

Eryfipelas, cafes of 280

Excitability, ufe of the cold bath to roufe 111

Excitement, on diminifhed 304

Experiments on fixed alkalies 93, 286

——— on the acids of urine, &c. 124

——— on the chemical agencies of electricity 190

——— galvanic 286

——— on oxalic acid 287

——— on the atmofpheric air 372

——— on the Chinefe raddifh 479

Eyes, on applying irritating fubftances to the 545

Fat, an occafion of pulmonary confumption 339

Fern, ufes of female 542

Fever, intermittent cured by calomel 15

——— cured by nafal hæmorrhage 53

——— ufe of the cold bath after 111

——— cafe of bilious 106

——— obfervations on 113

——— on the diagnofis between phrenitis and 179

——— as connected with inflammation, on 168

——— cafes of 220

——— ufe of mercury in typhus 315, 322

——— account of a typhus 366

Feverfew, ufes of the 457

Fibre, how medicine acts upon the living 365

Fire, a compofition which refifts 479

Fire-alarm, account of a 575

Fluor albus, on cantharides in 528

Foot, Dr. on rain water and dew 494

Fothergill's Dr. reports of difeafes 90, 186, 283, 368, 475

——— obfervations on the fore throat 177

Fowler's folution of arfenic, on 369

Fractures, on the treatment of 80

Fumitory, defcription and virtues of 71

Fungus hæmatodes, cafe of 215

——— obfervations on 429

Furze, defcription and ufes of 75

Gairdner, Dr. on cold water in the fmall-pox 273

Galvanic experiments 286

Galvanifm, on 325

Ganges, unhealthinefs of the 114

Gangrene, crural hernia, terminating in 501

Germination of feeds, effects of 371

Ginger, its efficacy in rheumatic gout 527

Glandular fwellings, on 363

Goldhagen, Dr. remarkable cafe of 171

Good, Mr. his medical oration 381

Gough, Mr. on dormice 383

Gout, cafe of 54

——— effects of cinchona bark in 427

——— on ginger in rheumatic 527

——— on cold water in 535

Graham's Mr. cafe of eruptions 524

Gum elaftic tube, advantage of 248

Hæmorrhage, intermittent, cured by nafal 53

——— efficacy of acetate of lead in uterine 69

——— of the lungs, cafe of 8

——— uterine, cafes of 9, 10

Hæmoptyfis, acetate of lead adminiftered in 70

Halliday, Dr. on epilepfy and worms 305

Hamilton, Dr. on purgative medicines 470

Hamm's, Dr. cafe of hydrocephalus 217

Harmattan, of the 115

Harleftonenfis, on the ufe of mercury in typhus 315

——— reply to 451

——— anfwer of 522

Harris's, Dr. cafe of hydrocephalus 22

——— cafe of phthifis pulmonalis 219

Harrifon, Dr. on ginger in rheumatic gout 527

Hawkins's Mr. mufeum, account of 575

Hawkridge, Mr. on diminifhed excitement 304

Hearing, on the theory of 385

Hernia, obfervations on 85

——— cafe of crural 501

——— on Mr. Bellott's cafe of 528

Home, Mr. the Copleian medal given to 191

Hooping cough, on the 91

——— cough, virtues of elecampane in 455

Hugo's, Mr. cafe of ruptured uterus 209

Hume, Mr. on fulphur as a vermifuge 382

Humphrey's, Dr. cafe of palfy, &c. 119

Hydrarthus, on 84

Hydrocephalus, fatal cafe of 22

——— obfervations on 91

——— fuccefsfully treated 217

Hydrogen, its union with metals 574

Hydrophobia, Dr. Turnbull's case of 117

———— observations on Dr. Moseley's case of 241

———— Mr. Broughton's case of 491

Hyslop Mr. receives the Jacksonian prize 575

Hysteralgia, case of 550

Hysteria, remedy for 354

Iceland moss, on the 34

Icteritia, observations on 189

Inflammation, on fever connected with 168

———— of the bowels, treatment of 183

———— of the lungs, dysphagia supervening in 248

Inflexibility of the articulations 4

Inoculation for the measles, on 153

———— popular view of vaccine 377

Insanity, on the causes of 94

Insect, fever occasioned by the bite of an 220

Intelligence, medical, 93, 190, 286, 381, 479, 574

Intermittent fever, cured by calomel 15

———— cured by nasal hæmorrhage 53

———— case of chronic *ib.*

Iron, effects of filings of 6

Jackson, Mr. on the cataract 101

Jenkinson, Mr. on chronic rheumatism 476

Jenner, Dr. letter to 556

Jennerian institution, report of the royal 327

Jenning's, Dr. case of fever 220

Jewel's, Mr. method of preparing calomel 382

Johnson, Dr. on an opinion of 475

Joints, on diseases of the 84

Joints, on dropsy of the *ib.*

Kellie's, Dr. case of chronic rheumatism 556

Kentish, Dr. in answer to Dr. Wood 417

———— reply to 518

———— address to 535

Kidney-vetch, description of 76

Kinglake, Dr. on the practice of 17, 54

———— on cinchona in gout 427

Lathyrus, description of the 77

Lawrence, Mr. on hernia 85

Lead, efficacy of acetate of 69

LECTURES ANNOUNCED. Mr. Brookes on surgery, &c. 95 Mr. Taunton on ditto, 95, 480. Dr. Clutterbuck on the theory and practice of physic, *ib.* Mr. Chevalier on surgery, *ib.* Dr. and Mr. Clarke on midwifery, 95, 288. Dr. Clough on ditto, 95, 480. Mr. Gunning on surgery, 96. Dr. Reid on the theory and practice of medicine, 96, 288. At St. Thomas's and Guy's Hospitals, 192. Mr. Moor on the teeth, *ib.* Mr. Young's Medical, 575. Mr. Carpue on surgery, 576

Leeches, injurious effects of 57

Le Gros, Mr. his case of burn 20

Letter, an extraordinary 428

Lichen islandicus, observations on the 34

Light of the pyrosoma atlanticum, on the 479

Lightning, case of palsy cured by 119

Lithotomy, on the operation of 271

Lime, effects of muriate of 6

London, account of diseases in 90, 92, 186, 189, 283, 285, 368, 370, 475, 477, 573

————, casualties and deaths in 96

———— medical society, meeting of 331

———— the proper school of physic 469

Lunatics, on the treatment of 545

Lungs, case of hæmorrhagy from the 8

———— dysphagia supervening an inflammation of the 248

———— remedy for disorders of the 455

Luxations, on the reduction of the 289

Lycopodium, description and uses of 541

Machel's, Mr. case of cancer 270

Macilwain, Mr. on the Ringwood cases 330

M'Dowell, Dr. on consumption 20

Madeira, on the accommodations at 19

Maidenhair, virtues of 544

Mammoth, discovery of a complete 287

Martin, Mr. on burns and scalds 17

Mease, Dr. on typhus 103

Measles, cases of 91, 185, 279, 471

———— cases of secondary diseases subsequent to 133

———— on inoculation for the 153

———— observations on 321

Medical reform, on 61, 462

———— consultations, on 204

———— police, plan of 238

———— society, meeting of the 381

———— practice, on American 414

Medicine, on abuses in 462

———— its action on the animal fibre 365

Melilot trefoil, description of 162

Menorrhagia, efficacy of cerusa acetata in chronic 11

Mercury, case of phthisis cured by 20

————, its use in typhus 109, 315

Midwifery, on the practice of 178

Milk, on the acid of 174

Milkwort, the virtues of 72

Miller, Dr. on sea sickness 295, 422

Mitchell, Dr. on the efficacy of the acetate of lead 69

Morality, on medical 2

Morbid body, dissection of a 160

Moſeley's caſe of hydrophobia, obſervations on 241

Moxa, preparation of the Japaneſe 357

Muſcles, on the action of the 289

Muſical ſounds, theory of 4[illegible]2

Naſal hæmorrhage, intermittent cured by 53

Necroſis, on 80

Nervous affection, caſe of 284

Noſological characteriſtics of diſeaſe 1

Nottingham, report of the hoſpital at 179

Oil, purgative virtue of olive 51

—— efficacy of it in cancer 142

—— of Chineſe raddiſh, uſe of the 479

Operation, improvement in ſurgical 481

Ophthalmia, obſervations on the 545

—— account of an 473

Opium, its effects counteracted by alkohol 497

—— its uſe in pulmonary conſumption 47

Oſmund royal, deſcription and uſes of 542

Oxalic acid, on 287

Palſy cured by lightning 119

Parliament, hints for the conſideration of 556

Patient, letter from a 350

Pemphigus gangrænoſus, on 344

Penis, extirpation of the cancer of the 270

Pepy's Mr. galvanic experiments 236

Percival Dr. account of 271

Phonics, theory of 385

Phrenitis, on the diagnoſis between fever and 179

Phthiſis pulmonalis, caſes of 219, 224

Phyſician, letter from a patient to his 350

Piles, remedy for 461

Piracy, refutation of a charge of literary 417

Plants, botanical deſcription of Britiſh 71, 162, 257, 353, 455, 538

—— on the vegetation of 371

—— on the phyſiology of 381

Plica polonica, remedy for the 540

Pneumonia, effects of ceruſſa acetata in 9

Police, plan of medical 237

Pollock, Mr. on the ſubmerſion of ſwallows 351

Portland powder, on the 356

Practice, on American medical 414

Price, Mr. on anti-vacciniſt candour 426

Prouſt, Mr. on the cauſe of inſanity 94

Publications, new medical 192, 288, 384, 576

Pulmonary complaints, prevalence of 472

Pulmonary conſumption cured by ſalivation 20, 219, 224, 506

—— caſes of 47

Pulmonary conſumption, on the treatment of 263

—— uſe of emetics in 337

—— uſe of Chineſe raddiſh in 479

—— on ſtimulants in 510

—— uſe of digitalis in 511, 515

Purgative, efficacy of olive oil as a 51

Purgative medicines, obſervations on 470

Pyroſoma atlanticum, on the light of the 479

Quack, extraordinary letter of a 428

Quarantines, obſervations on 113

Raddiſh, virtue of the oil of Chineſe 479

Rain-water, noxious quality of 494

Reform, on medical 61, 462

Reſpiration of animals, theory of 371

—— remarks on 547

Rheumatic affections, on 187

—— on the cinchona bark in 427

—— on arſenic in chronic 476

—— gout, on ginger in 527

Ring's, Mr. reply to Mr. Davies 53

Ringwood caſes, account of the 193, 327

Roberton's, Mr. report of the diſeaſes of Edinburgh 182, 273, 278, 361, 470, 569

—— plan of medical police 237

Robertſon's Dr. colloquia anatomica 178

Rupture of the uterus, caſe of 209

Ruſh's Dr. caſes of pulmonary conſumption 47

—— on the effects of ſalivation and ſtimulants 506

Rutter's Dr caſe of hyſteralgia 550

Salivation, its efficacy in conſumption 20, 224, 506

Sanguiferous ſyſtem, on the exceſſive action of the 436

Saunders, Dr. on pulmonary conſumption 263

Scalds, on the treatment of 17, 53, 229, 518

Scrophulous affections, remedy for 74

Scurvy, remedy for the 262

Sea ſickneſs, obſervations on 295, 422

Seaſons attending epidemics, on 317

Seeds, on the germination of 371

Sharkey, Dr. on the uſe of emetics 337

Shephard, Mr. on alkohol 497

Skaiters, on accidents occurring to 283

Small-pox, on the prevention of the 186

—— on the uſe of cold water in 272

—— caſes of 150, 286, 368

—— hoſpital, on the ſtate of the 557

Smith's, Dr. caſe of diſeaſe in the ſtomach 157

Sounds, ſketch of a theory of 407

Spence, Dr. on digitalis in conſumption 511

Spleenwort, virtues of the 543

Sporadic bad air, on 201

Stimulants in conſumption, on 510

Stokes, Dr. on pemphigus gangrænoſus 344

Stomach, caſe of diſeaſe in the 157

——— of complaints in the 474

Stone, remedy for the 361

Strabiſmus, obſervations on 313

Stringham, Dr. on digitalis purpurea 420

Stromeyer's chemical arrangements and inveſtigations 574

Stuart, Dr. on the injurious effects of leeches 57

Sulphur as a vermifuge, on 382

Surgical operation, firſt principle of 491

Swallows, on the ſubmerſion of 351

Sweat, analyſis of 124

Sydenham on the ſmall-pox 150

Syroc, on the 116

Tanſy, deſcription and uſes of the 353

Terebinthinate plan of treating ſcalds, on the 17

Tetanus, effects of mercury in 111

——— on 243

——— caſe of 439

Thenard, Mr. on the analyſis of ſweat, &c. 124

Trefoil, deſcription and uſes of 163

Turnbull's Dr. caſe of hydrophobia 117

Turpentine in ſcalds, on 54

Typhus, good effects of mercury in 109

——— obſervations on the uſe of mercury in 315

——— on cold water in 321

Ulcer, effects of argentum nitratum in 97

Urea not cryſtallizable 131

Urethra, mal-conformation of the 123

Urine, on the acid of 124

——— caſe of ſuppreſſion of 227

Uterine hæmorrhage, efficacy of acetate of lead in 9, 10, 49

Uterus, caſe of rupture of the 290

Vaccination, obſervations on 54

——— report on the ſuppoſed failures of 327

——— popular view of 377

——— obſervations on 434

——— on the conduct of the enemies to 442

Valentine, Dr. on the actual cautery 445

Varley, Mr. on the remarks of 325

Vaughan, Dr. on the efficacy of olive oil 51

——— his caſe of intermittent 53

——— caſe of ſcald *ib.*

Vegetables, on clearing them of inſects 382

Vegetation of plants, on the 371

Vermifuge, on ſulphur as a 382

Vertigo, obſervations on 299

Vetch, Dr. on ophthalmia 545

Viſcera, remedies for obſtructions of the 72, 167

Volcano in Wales 382

Wadſworth's, Dr. caſe of diſſection 160

Wales, volcanic remains in 383

Walker, Mr. his theory of phonics, &c. 385

——— firſt principle of ſurgical operation 481

———, Dr. on vaccination 434

Water, cold, its efficacy in rouſing excitability 111

——— in ſmall pox 272

——— in typhus 175, 321

——— in gout, on 535

——— compoſition to reſiſt the action of 479

Weaver, Mr. on the uſe of arſenic in eruptions 206

Weſtcott, Mr. on the Ringwood caſes 330

Wilſon's, Dr. caſes of intermittent fever cured by calomel 15

——— on fever and phrenitis 179

Winds, on noxious 116

Wood, Dr. on the treatment of burns 229

——— anſwer to 417

——— his reply 518

———, Mr. on injury to the canal of the urethra 270

Worms, remedies for 164, 354

——— caſe of 305

——— uſe of ſulphur in 382

Wormwood, uſes of 355

Wright, Dr. on the uſe of cold water in ſmall-pox 272

Yarrow, medical virtues of 460

Yaws, minutes of a caſe of 122

Young, Mr. on the practice of adheſion 563

